# Supplementary material for: Biogenesis of a bacterial metabolosome for propanediol utilization
Source: Nat Commun. 2022 May 25;13:2920. doi: 10.1038/s41467-022-30608-w (PMC9132943; doi:10.1038/s41467-022-30608-w)
Supplement: Supplementary file 1 — Supplementary Information [file 41467_2022_30608_MOESM1_ESM.pdf]

Supplementary Information

for

Yang et al., “Biogenesis of a bacterial metabolosome for propanediol utilization”

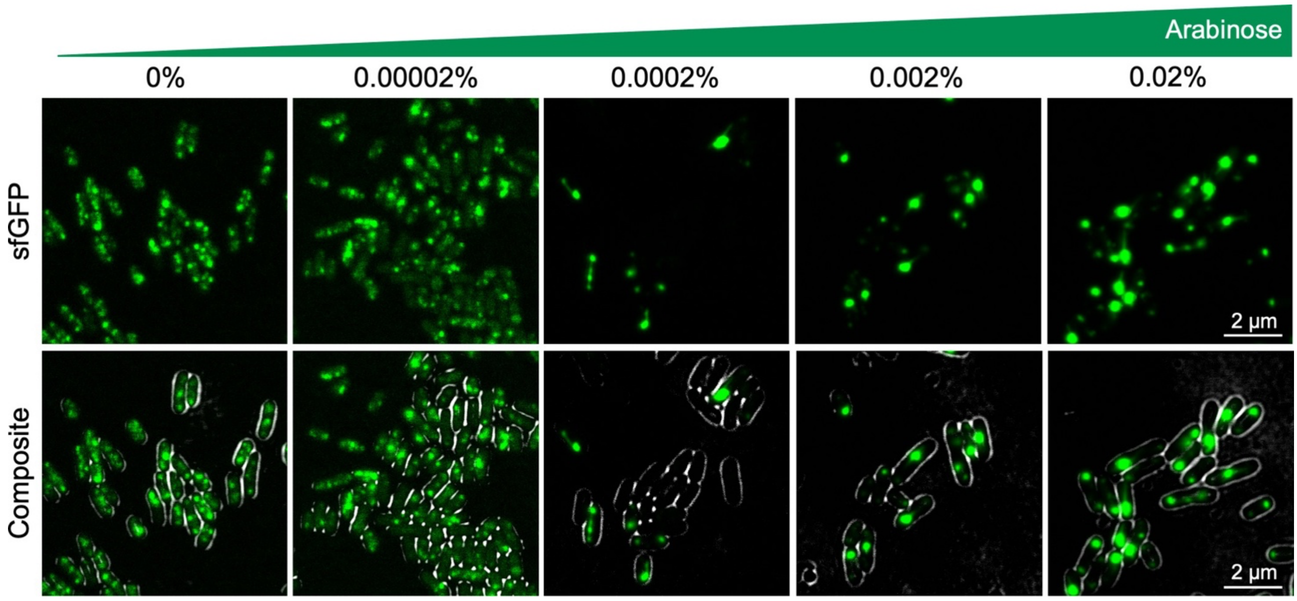

Supplementary Fig. 1. Fluorescence images show WT LT2 carrying pBAD-*pduA-sfGFP* grown in MIM+1,2-PD media at various arabinose concentrations (g·mL^{-1}). Before fluorescence imaging, *Salmonella* cells were grown in a 2-mL Eppendorf tube shaken horizontally and aerobically at 37°C at 220 rpm until OD_{600} reaching 1.0-1.2.

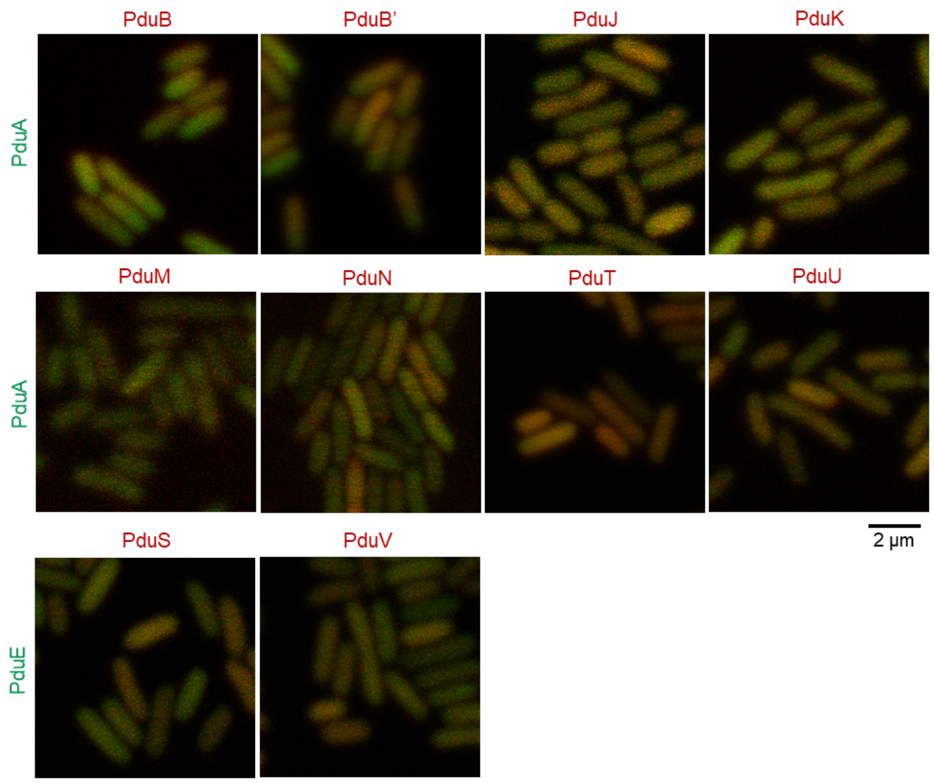

Supplementary Fig. 2. Pdu BMCs are not formed in the absence of 1,2-PD, as revealed by fluorescence images of the WT LT2 cells carrying pBAD (expressing fluorescently tagged PduA/E/B/B'/J/K/M/N/T/U/S/V proteins) grown in MIM-1,2-PD media. Green represents the fluorescence of Pdu proteins tagged with sfGFP and red represents the fluorescence of proteins tagged with mCherry. Before fluorescence imaging, *Salmonella* cells were grown in a 2-mL Eppendorf tube shaken horizontally and aerobically at 37°C at 220 rpm until OD_{600} reaching 1.0-1.2.

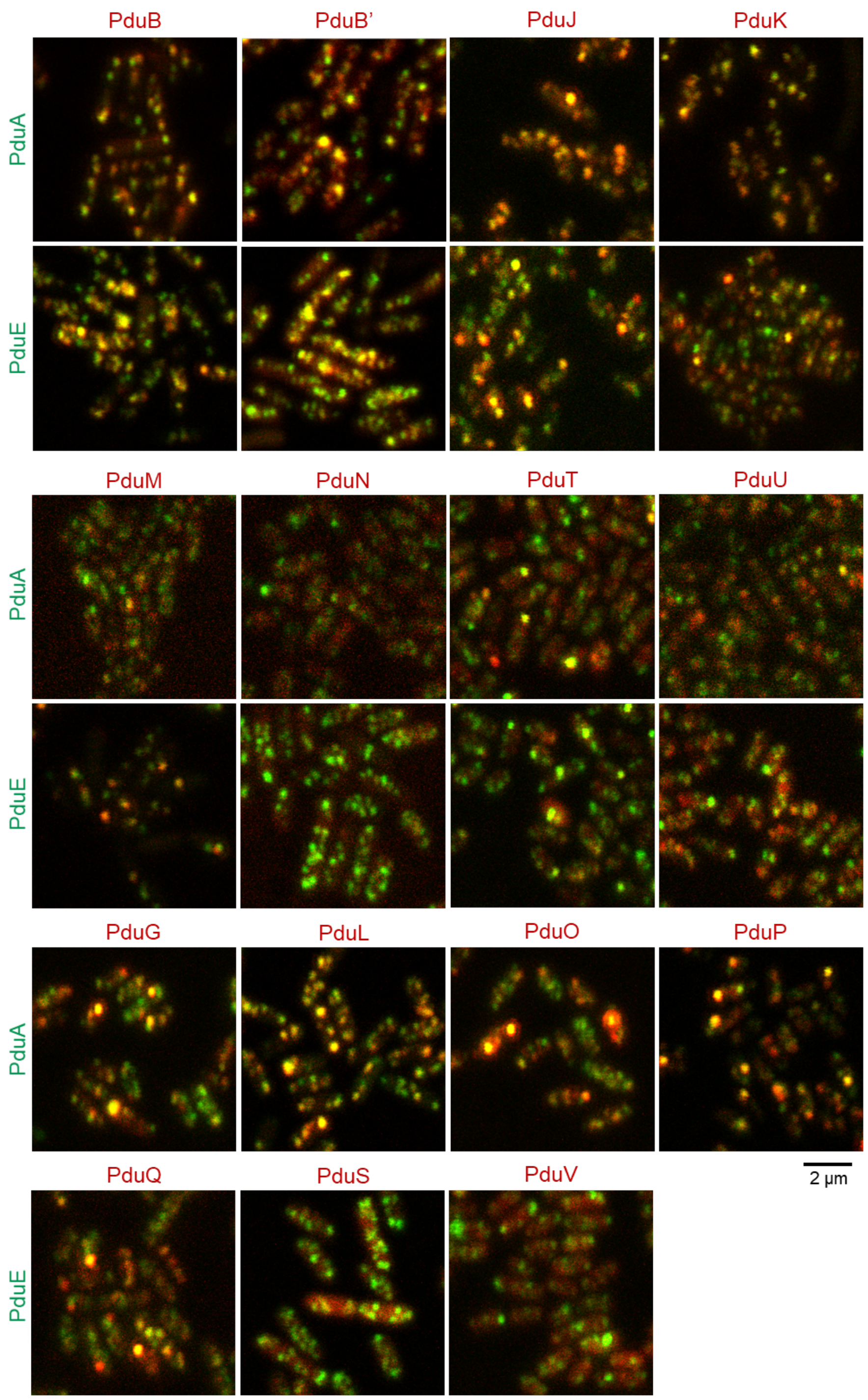

Supplementary Fig. 3. Pdu BMCs are formed in the presence of 1,2-PD. Fluorescence images show WT LT2 carrying pBAD (expressing fluorescently tagged Pdu proteins) grown in MIM+1,2-PD media. Before fluorescence imaging, *Salmonella* cells were grown in a 2-mL Eppendorf tube shaken horizontally and aerobically at 37°C at 220 rpm until OD_{600} reaching 1.0-1.2. Green represents the fluorescence of Pdu proteins tagged with sfGFP; red represents the fluorescence of proteins tagged with mCherry.

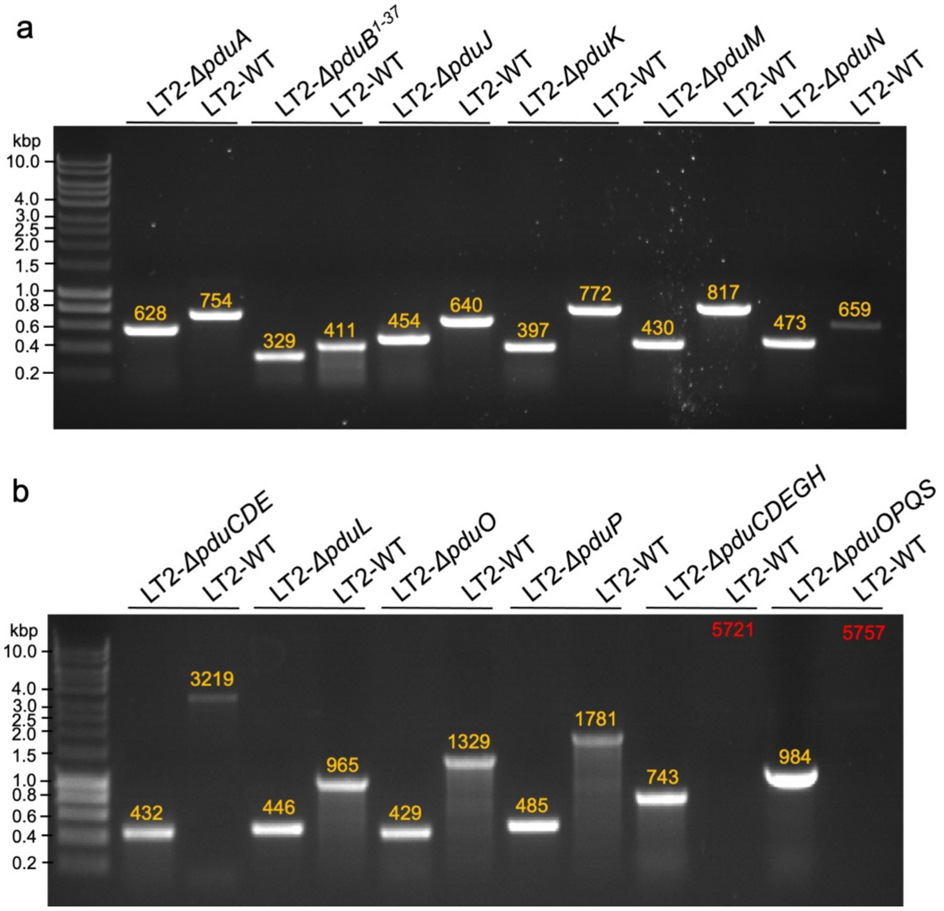

Supplementary Fig. 4. PCR-based confirmation of *S.* Typhimurium LT2 gene deletion mutants. (**a**) and (**b**) correspond to shell and catalytic gene deletions, respectively. The sizes of the PCR products are indicated (bp, yellow). No band was detected in two WT strains due to the expected size (red) being too long to synthesize under these amplification conditions.

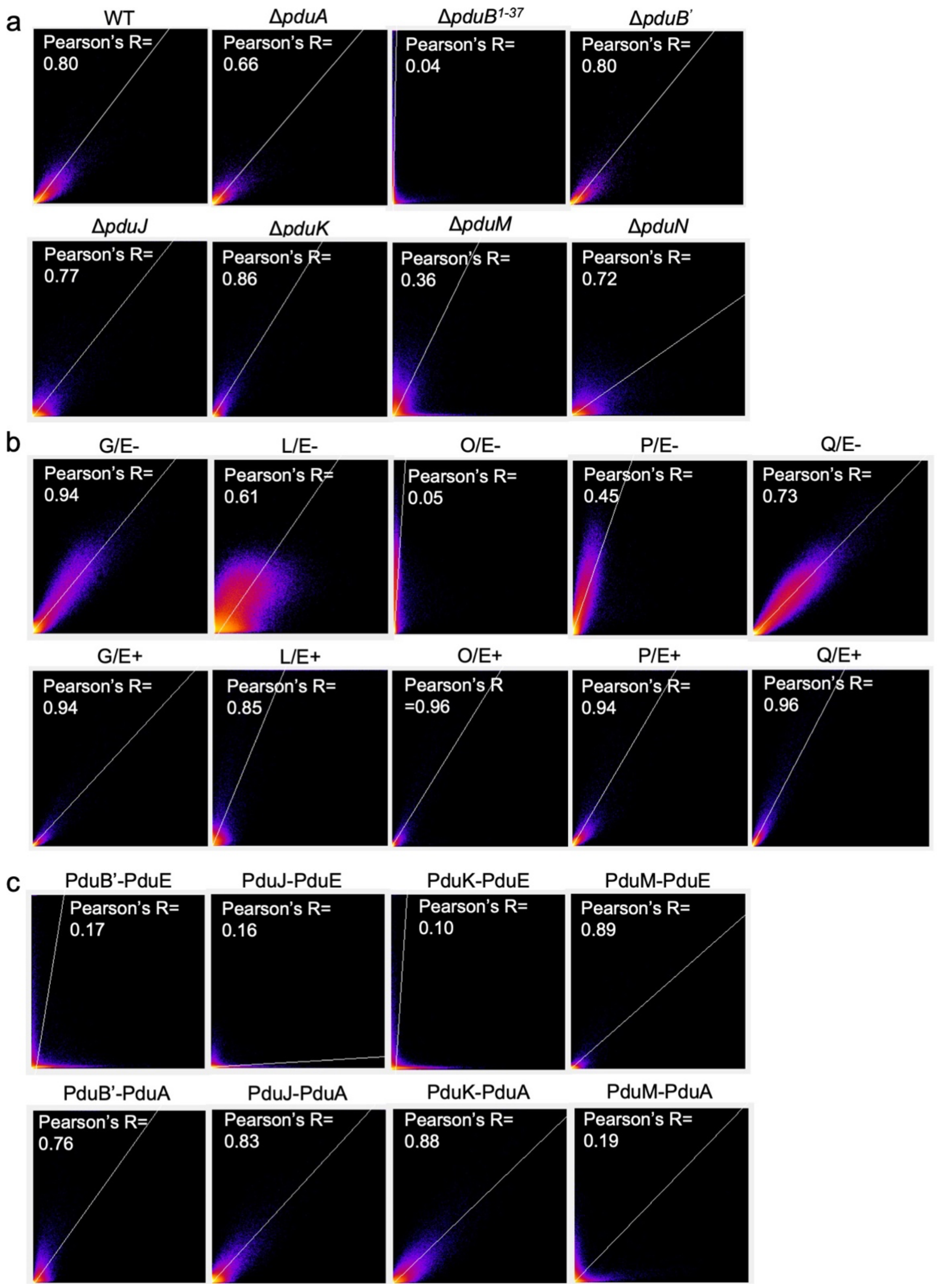

Supplementary Fig. 5. Scatterplot of colocalization analysis of data from Figs. 2 and 3. (**a-c**) are representative scatterplots of Figs. 2b, 3b, and 3f, respectively. Scatterplots are generated by plotting the intensity value of each pixel of mCherry along the x-axis and the intensity value of the same pixel location of sfGFP on the y-axis using Coloc2 plugins in ImageJ. The scatterplots describe the relationship between the fluorescent signals. If the dots on the diagram appear as a cloud clustered on a line, a strong colocalization is indicated, and the Pearson's R is close to 1. If the scattered distribution of the pixels is close to both axes, a mutual exclusion is indicated, and the Pearson's R is near zero. Note: in panel (**b**), the first capital letter of the name is the name of the Pdu protein tagged with mCherry and the second capital letter of the name is the name of the Pdu protein tagged with sfGFP. '+' and '-' represents the presence and absence of 1,2-PD in the growth media, respectively. For example: 'G/E+' stands for PduG-mCherry/PduE-sfGFP in presence of 1,2-PD.

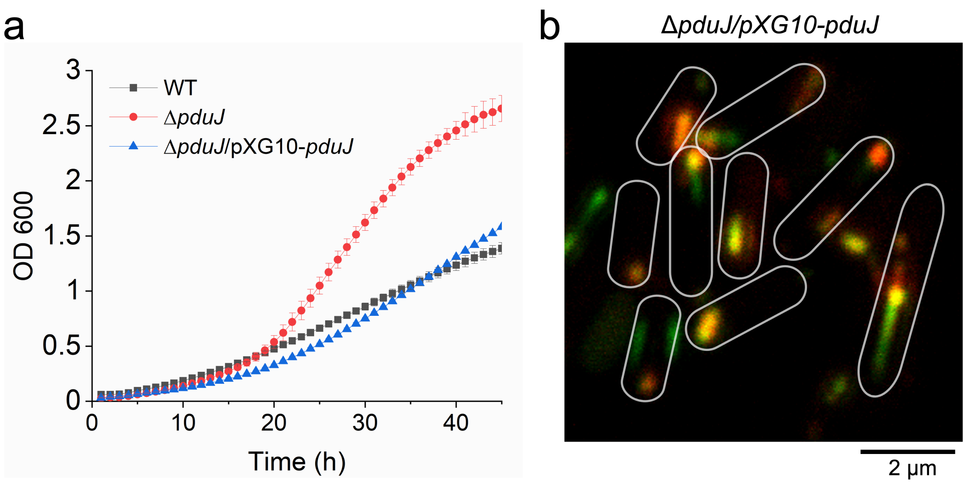

Supplementary Fig. 6. Complementation of PduJ. (**a**) Growth curves of WT, Δ*pduJ*, and the Δ*pduJ* strain that expressed the PduJ protein using a pXG10 plasmid, in the NCE medium (containing 0.3 mM each of leucine, isoleucine, threonine, and valine, 50 µM ferric citrate) with 0.6% 1,2-PD and limiting B_{12} (20 nM). Data are represented as mean ± SD. $n = 4$, n represents the number of biologically independent experiments. (**b**) Confocal microscopic image of the Δ*pduJ* strain expressing PduJ from a pXG10 plasmid grown in MIM+1,2-PD medium.

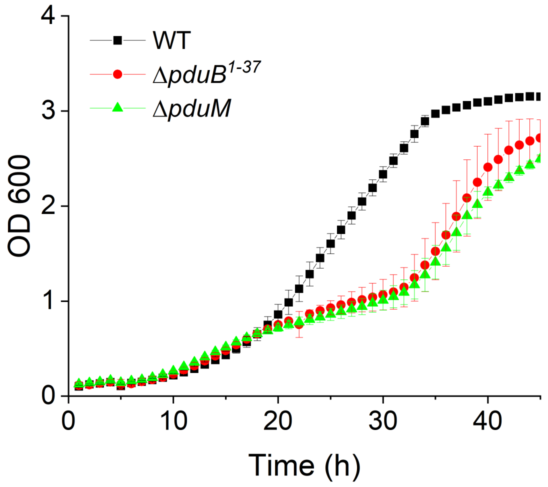

Supplementary Fig. 7. Growth curves of the *S.* Typhimurium LT2 WT and the Δ*pduB*$^{1\text{-}37}$ and Δ*pduM* mutants in the presence of 1,2-PD (0.6%, v/v) with saturating vitamin B_{12} (150 nM). The medium was the NCE medium (supplemented with 0.6% (v/v) 1,2-PD and 150 nM vitamin B_{12}). Growth curves were measured on a Growth Profiler 960 (EnzyScreen) under aerobic conditions. Data are represented as mean ± SD. $n = 3$, n represents the number of biologically independent experiments.

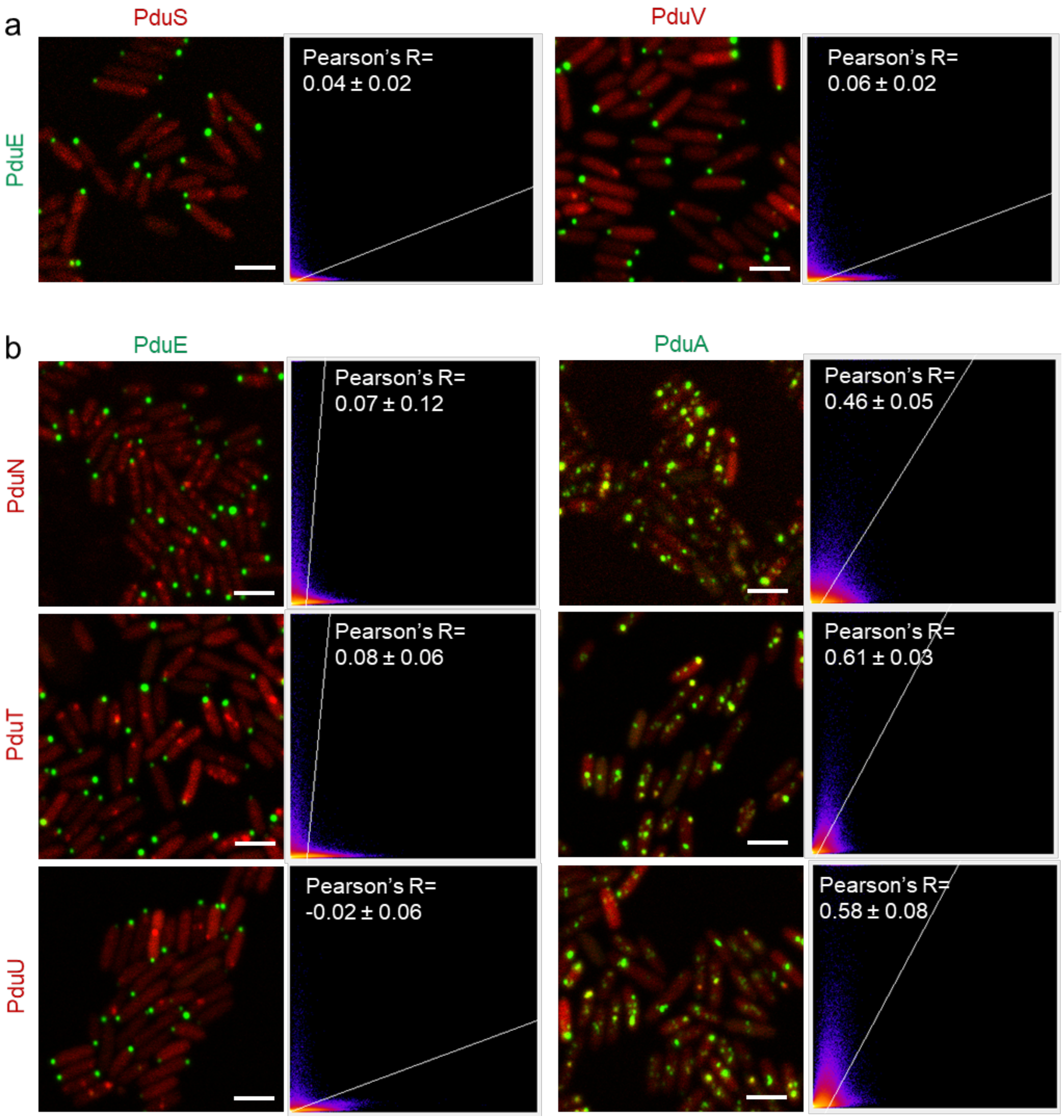

Supplementary Fig. 8. Minor Pdu enzymes (PduS/V) and minor Pdu shell proteins (PduN/T/U) are not colocalized with PduE (cargo). (**a**) Fluorescence imaging of Δ*pduB*$^{1\text{-}37}$ expressing PduS-mCherry/PduE-sfGFP and PduV-mCherry/PduE-sfGFP (grown in MIM+1,2-PD media), and scatterplots of colocalization analysis. (**b**) Fluorescence imaging on Δ*pduB*$^{1\text{-}37}$ expressing minor shell protein (PduN, PduT and PduU) tagged with mCherry and PduE-sfGFP or PduA-sfGFP (grown in MIM+1,2-PD media), and scatterplots of colocalization analysis. Before fluorescence imaging, *Salmonella* cells were grown in a 2-mL Eppendorf tube shaken horizontally and aerobically at 37°C at 220 rpm until OD_{600} reaching 1.0-1.2. For Pearson's R values calculation, n = 20, n represents the number of cells. Data are represented as mean ± SD.

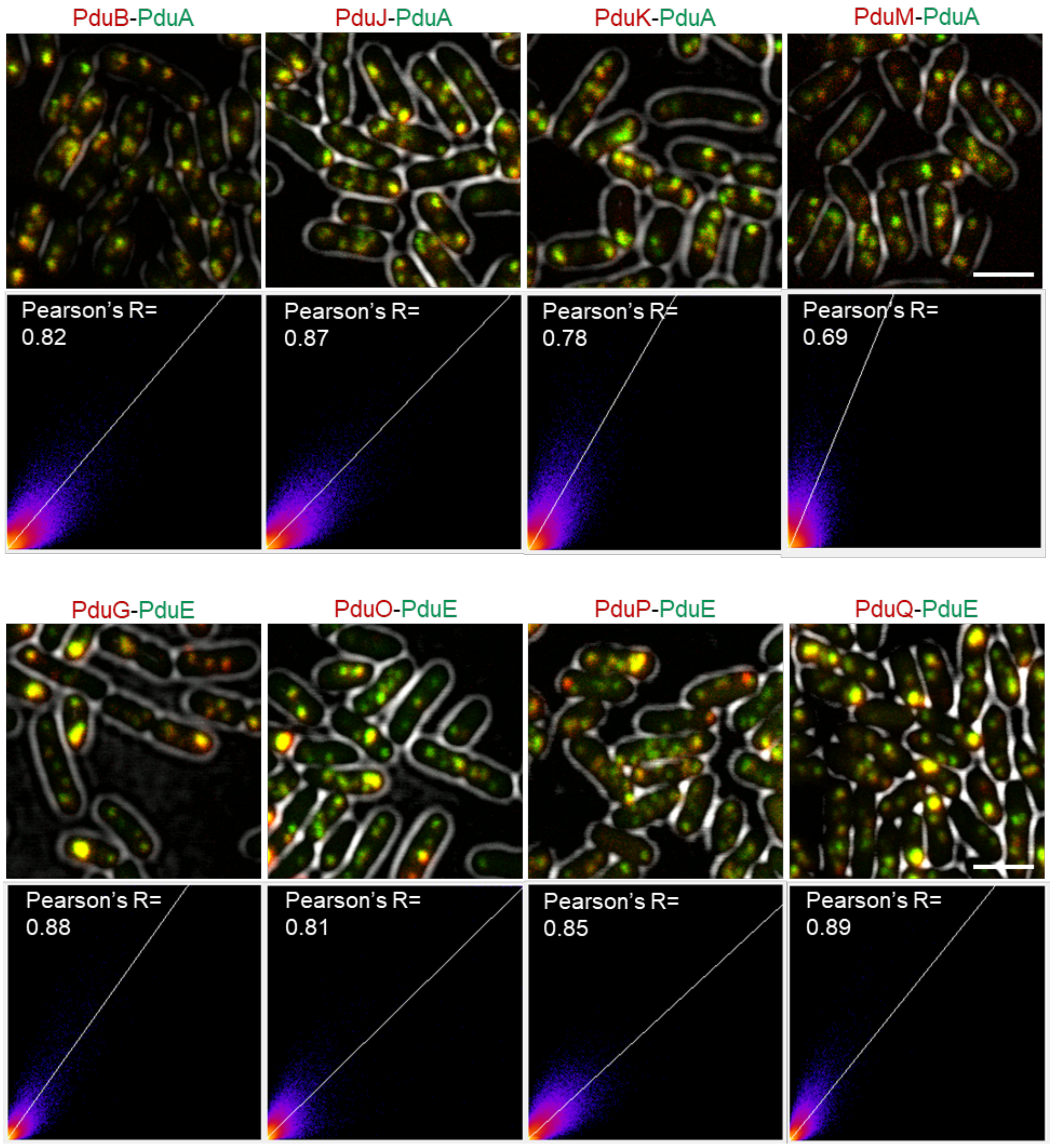

Supplementary Fig. 9. PduB' is not essential for assembly of the Pdu MCP. Fluorescence imaging shows in presence of Pdu MCPs in Δ*pduB'* grown in MIM+1,2-PD media. Before fluorescence imaging, *Salmonella* cells were grown in a 2-mL Eppendorf tube shaken horizontally and aerobically at 37°C at 220 rpm until OD_{600} reaching 1.0-1.2. Green represents the fluorescence of Pdu proteins tagged with sfGFP; red represents the fluorescence of proteins tagged with mCherry. Scatterplots are generated by plotting the intensity value of each pixel of mCherry along the x-axis and the intensity value of the same pixel location of sfGFP on the y-axis using Coloc2 plugins in ImageJ.

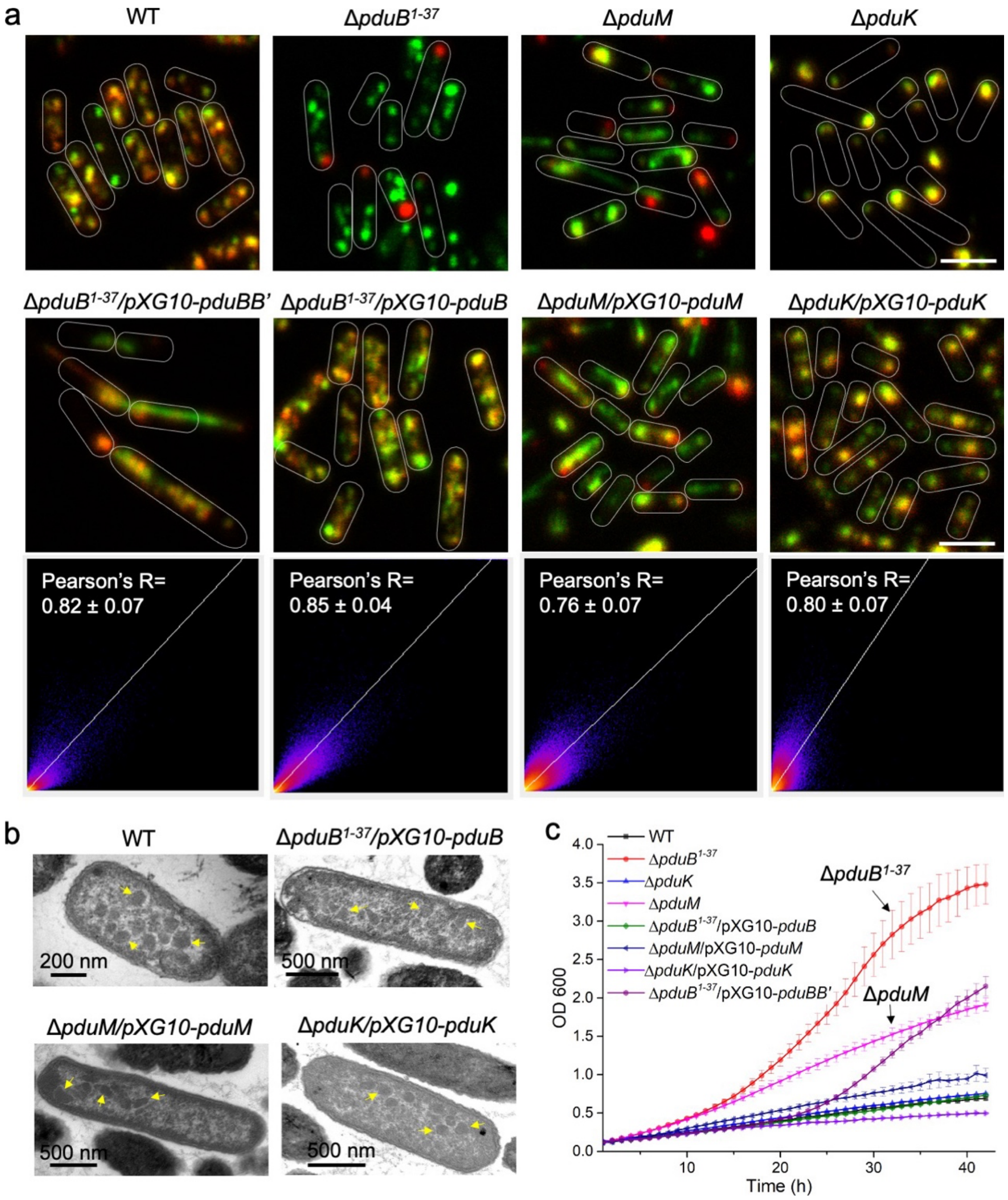

Supplementary Fig. 10. Successful complementation of PduB and PduK, and partial complementation of PduM. (**a**) Fluorescence imaging on gene deletion mutants expressing deleted proteins from pXG10 plasmid grown in MIM+1,2-PD media, and scatterplots of colocalization analysis. Before fluorescence imaging, *Salmonella* cells were grown in a 2-mL Eppendorf tube shaken horizontally and aerobically at 37°C at 220 rpm until OD_{600} reaching 1.0-1.2. For Pearson's R values calculation, n = 20, n represents the number of cells. Data are represented as mean ± SD. (**b**) EM of WT and gene deletion mutants expressing deleted proteins from pXG10 plasmid grown in MIM+1,2-PD media. The Pdu MCP structures are indicated with yellow arrows. Before EM sample preparation, 10 mL *Salmonella* cells were grown aerobically in 50 mL Falcon tubes at 37°C at 220 rpm until OD_{600} reaching 1.0-1.2. (**c**) Growth curves of gene deletion mutants carrying pXG10-based plasmids expressing the appropriate Pdu protein, during grown in 1,2-PD with limiting B_{12} (20 nM). The medium was NCE medium (containing 0.6% 1,2-PD; 0.3 mM each of leucine, isoleucine, threonine, and valine; 50 μM ferric citrate; 20 nM vitamin B_{12}). Data are represented as mean ± SD. n = 4, n represents the number of biologically independent experiments.

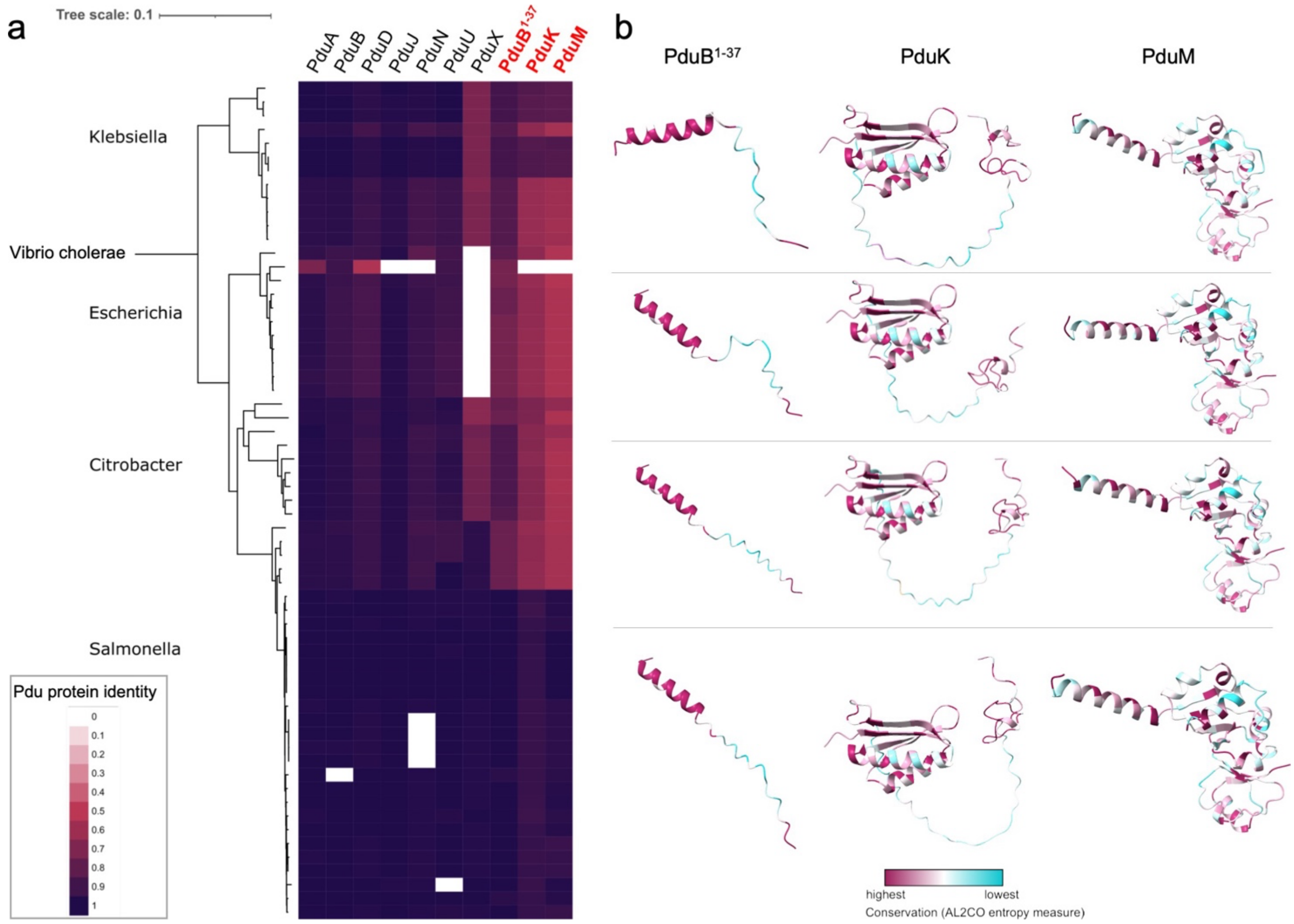

Supplementary Fig. 11. Similarities between the PduB$^{1\text{-}37}$, PduK and PduM proteins from four bacterial genera (*Klebsiella*, *Escherichia*, *Citrobacter,* and *Salmonella*) at the amino acid and structural levels. (**a**) The phylogenetic tree was made from an alignment of 327 core genes from 61 bacterial genomes (see Source Data). The tree was rooted on the *Vibrio cholerae* MS6 genome as an outgroup. The heatmap shows the similarity of ten Pdu protein sequences of the 61 genomes comparing to *Salmonella* Typhimurium LT2. The levels of conservation of PduB$^{1\text{-}37}$, PduK, and PduM are mirrored by the conservation of other structural or essential Pdu proteins (PduA/B/D/J/N/U) encoded by the *pdu* operon. (**b**) The high structural similarity of proteins from different genera is inferred from AlphaFold 2 structural prediction. The structures are coloured by the conservation values of each residue in the alignment of 61 isolates. Four rows of the predicted structures are from four bacterial genera (from top to bottom, *Klebsiella*, *Escherichia*, *Citrobacter,* and *Salmonella*, respectively).

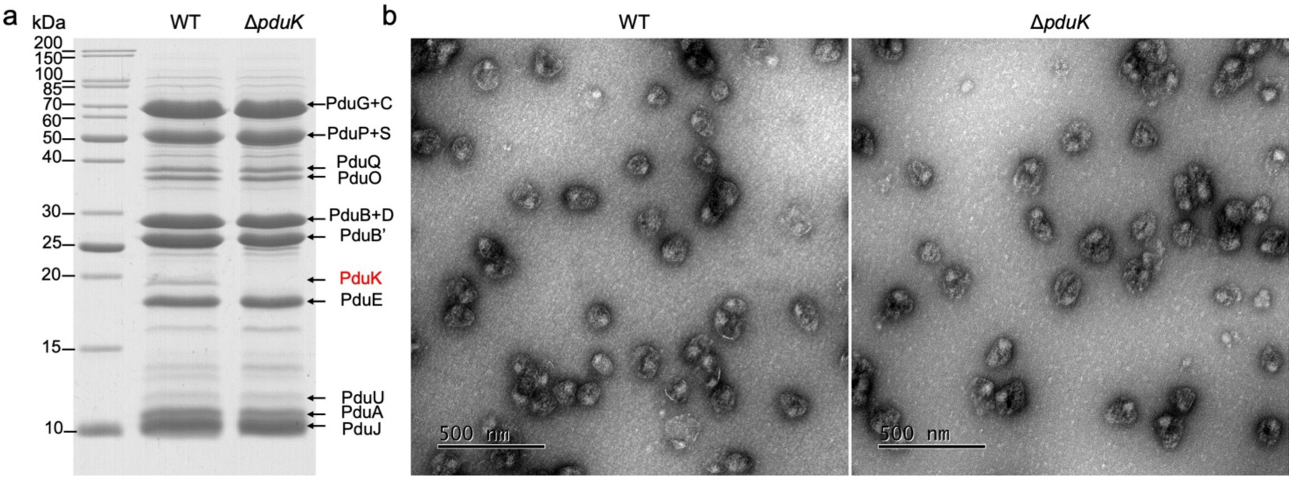

Supplementary Fig. 12. Isolation of Pdu BMCs from the *S*. Typhimurium LT2 WT and Δ*pduK* mutant. (**a**) SDS-PAGE of isolated Pdu BMCs from the WT and Δ*pduK* cells. The Pdu BMCs were isolated from 400 mL cells grown aerobically in 1L flask in the MIM+1,2-PD medium (OD_{600} = 1.0-1.2). (**b**) Negative-staining EM images of isolated Pdu MCPs from the WT and Δ*pduK* cells.

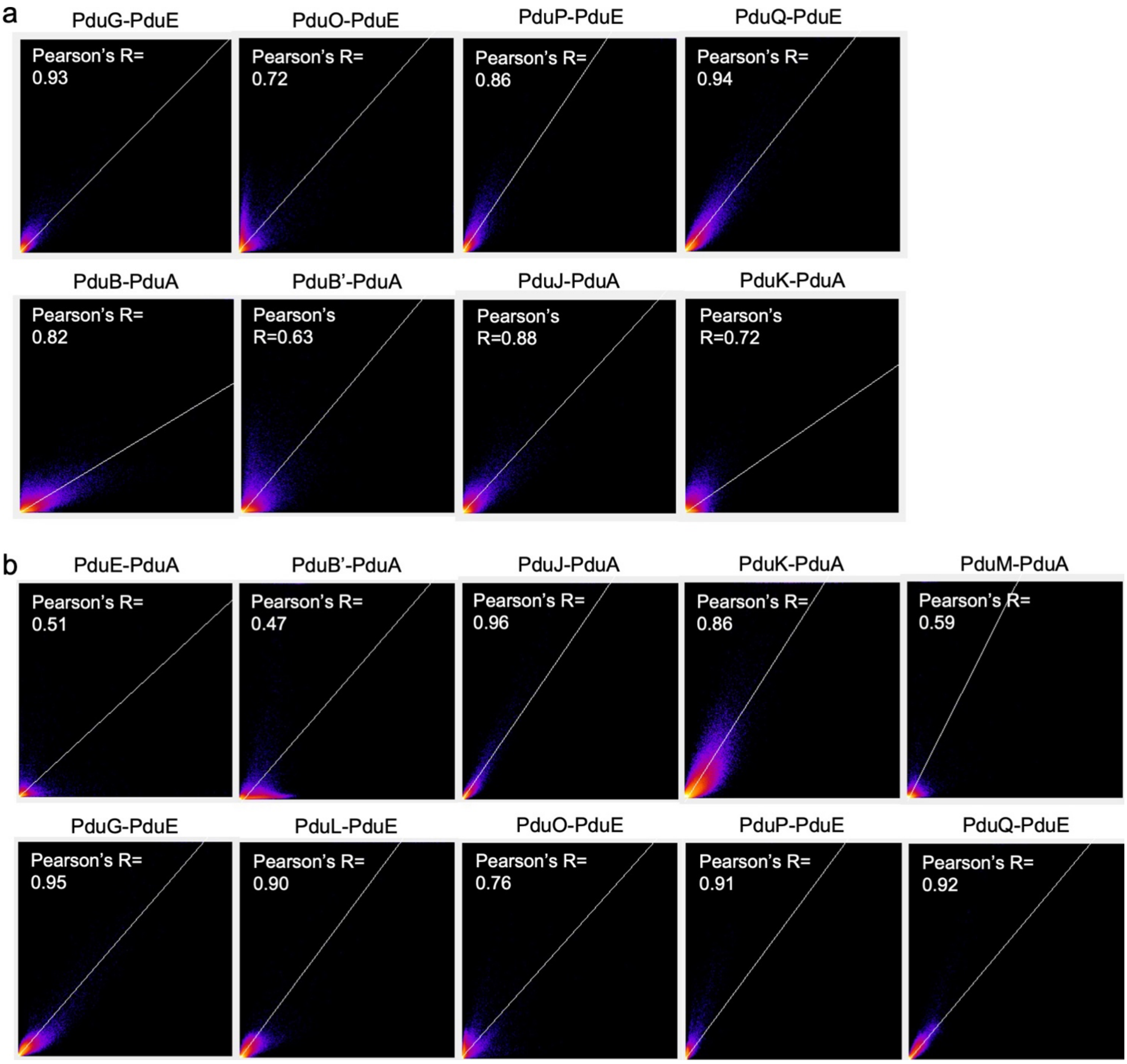

Supplementary Fig. 13. Scatterplot of colocalization analysis of data from Fig. 4. (**a**) and (**b**) are representative scatterplots of Fig. 4B and 4F, respectively.

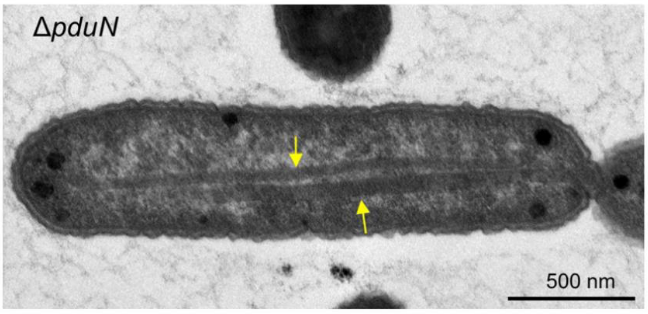

Supplementary Fig. 14. Thin-section EM of the Δ*pduN* strain revealing the elongated Pdu BMC structures (arrows) grown in the MIM+1,2-PD media. Before EM sample preparation, 10 mL *Salmonella* cells were grown aerobically in 50 mL Falcon tubes at 37°C at 220 rpm until OD_{600} reaching 1.0-1.2.

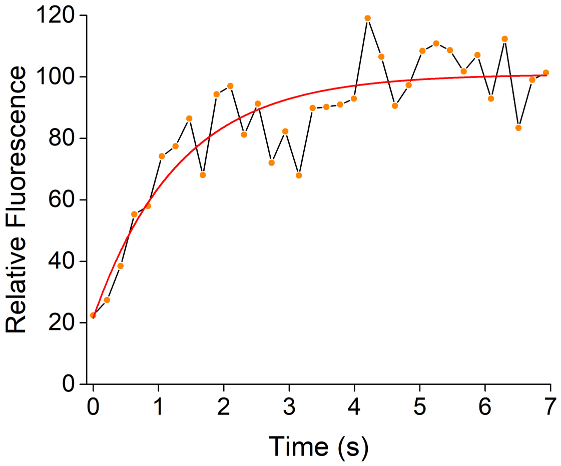

Supplementary Fig. 15. Time course of fluorescence recovery of bleached regions of free PduGH-sfGFP proteins. The y-axis indicates fluorescence values relative to fluorescence intensity of the selected region prior to bleaching. The recovery of sfGFP fluorescence is shown as circles which were fitted to an exponential function.

Supplementary Table 1. Strains of *S.* Typhimurium LT2 derivatives and plasmids. Relevant antibiotic resistances are indicated by ^{R}: Ap, ampicillin; Km, kanamycin; Cm, chloramphenicol; Gm, gentamicin; Tc, tetracycline.

Strains/Plasmids	Description	Origin
**LT2 derivatives:**		
LT2	LT2, WT	[1]
LT2-Δ*pduA*	Δ*pduA*	This study
LT2-*pduA*-sf*GFP*	LT2 derivative with PduA fused with sfGFP	This study
LT2-Δ*pduB*$^{1-37}$	The N-terminus that differentiate PduB and PduB' was deleted	This study
LT2-Δ*pduB*$^{'}$	The start codon for PduB' is replaced by GCT (alanine)	This study
LT2-Δ*pduJ*	Δ*pduJ*	This study
LT2-Δ*pduK*	Δ*pduK*	This study
LT2-Δ*pduM*	Δ*pduM*	This study
LT2-Δ*pduN*	Δ*pduN*	This study
LT2-Δ*pduCDE*	Δ*pduCDE*	This study
LT2-Δ*pduOPQS*	Δ*pduOPQS*	This study
LT2-Δ*pduB*$^{1-37}$/Δ*pduK*	Δ*pduB*$^{1-37}$ and Δ*pduK* double mutant	This study
LT2-Δ*pduB*$^{1-37}$/Δ*pduCDE*	Δ*pduB*$^{1-37}$ and Δ*pduCDE* double mutant	This study
LT2-Δ*pduB*$^{1-37}$/Δ*pduOPQS*	Δ*pduB*$^{1-37}$ and Δ*pduOPQS* double mutant	This study
***E. coli* derivatives:**		
E. coli S17-1 λ*pir*	*pro thi hsdR recA* chromosome::RP4-2 Tc::Mu Km::Tn7/λ*pir*; Tp^R, Sm^R	[2]
**Plasmids:**		
pKD4	*aph*-cassette template plasmid; Km^R	[3]
pKD3	Template for amplification of Cm^R cassette; Cm^R, Ap^R	[3]
pCP20	Plasmid carrying the Flp recombinase to remove kanamycin resistance from pKD13 derived resistance cassette insertions; Ap^R	[3]
pSIM5-*tet*	λ Red recombination plasmid, temperature-inducible; Tc^R	[4]
pEMG	Suicide plasmid; Km^R	[5]
pEMG-pduB1-37_del	pEMG bearing a 1kb EcoRI-BamHI insert for deleting region pduB1-37; Km^R	This study
pEMG-pduB M38A	pEMG bearing a 1kb EcoRI-BamHI insert for pduB M38A mutation; Km^R	This study
pSW-2	Plasmid for m-toluate–inducible expression of the I-SceI enzyme; Gm^R	[5]
pBAD/*Myc*-His	Vector for dose-dependent expression of recombinant proteins; Ap^R	Invitrogen
pBAD-*EA*	*pduE::mCherry-pduA::sfGFP* cloned into pBAD/*Myc*-His at NcoI and HindIII sites; Ap^R	This study
pBAD-*BA*	*pduB::mCherry-pduA::sfGFP* cloned into pBAD/*Myc*-His at NcoI and HindIII sites; Ap^R	This study
pBAD-*B'A*	*pduB'::mCherry-pduA::sfGFP* cloned into pBAD/*Myc*-His at NcoI and HindIII sites; Ap^R	This study
pBAD-*JA*	*pduJ::mCherry-pduA::sfGFP* cloned into pBAD/*Myc*-His at NcoI and HindIII sites; Ap^R	This study
pBAD-*KA*	*pduK::mCherry-pduA::sfGFP* cloned into pBAD/*Myc*-His at NcoI and HindIII sites; Ap^R	This study

pBAD-*MA*	*pduM::mCherry-pduA::sfGFP* cloned into pBAD/*Myc*-His at NcoI and HindIII sites; Ap^R	This study
pBAD-*NA*	*pduN::mCherry-pduA::sfGFP* cloned into pBAD/*Myc*-His at NcoI and HindIII sites; Ap^R	This study
pBAD-*TA*	*pduT::mCherry-pduA::sfGFP* cloned into pBAD/*Myc*-His at NcoI and HindIII sites; Ap^R	This study
pBAD-*UA*	*pduU::mCherry-pduA::sfGFP* cloned into pBAD/*Myc*-His at NcoI and HindIII sites; Ap^R	This study
pBAD-*BE*	*pduB::mCherry-pduE::sfGFP* cloned into pBAD/*Myc*-His at NcoI and HindIII sites; Ap^R	This study
pBAD-*B'E*	*PduB'::mCherry-pduE::sfGFP* cloned into pBAD/*Myc*-His at NcoI and HindIII sites; Ap^R	This study
pBAD-*JE*	*pduJ::mCherry-pduE::sfGFP* cloned into pBAD/*Myc*-His at NcoI and HindIII sites; Ap^R	This study
pBAD-*KE*	*pduK::mCherry-pduE::sfGFP* cloned into pBAD/*Myc*-His at NcoI and HindIII sites; Ap^R	This study
pBAD-*ME*	*pduM::mCherry-pduE::sfGFP* cloned into pBAD/*Myc*-His at NcoI and HindIII sites; Ap^R	This study
pBAD-*NE*	*pduN::mCherry-pduE::sfGFP* cloned into pBAD/*Myc*-His at NcoI and HindIII sites; Ap^R	This study
pBAD-*TE*	*pduT::mCherry-pduE::sfGFP* cloned into pBAD/*Myc*-His at NcoI and HindIII sites; Ap^R	This study
pBAD-*UE*	*pduU::mCherry-pduE::sfGFP* cloned into pBAD/*Myc*-His at NcoI and HindIII sites; Ap^R	This study
pBAD-*GE*	*pduG::mCherry-pduE::sfGFP* cloned into pBAD/*Myc*-His at NcoI and HindIII sites; Ap^R	This study
pBAD-*LE*	*pduL::mCherry-pduE::sfGFP* cloned into pBAD/*Myc*-His at NcoI and HindIII sites; Ap^R	This study
pBAD-*OE*	*pduO::mCherry-pduE::sfGFP* cloned into pBAD/*Myc*-His at NcoI and HindIII sites; Ap^R	This study
pBAD-*PE*	*pduP::mCherry-pduE::sfGFP* cloned into pBAD/*Myc*-His at NcoI and HindIII sites; Ap^R	This study
pBAD-*QE*	*pduQ::mCherry-pduE::sfGFP* cloned into pBAD/*Myc*-His at NcoI and HindIII sites; Ap^R	This study
pBAD-*SE*	*pduS::mCherry-pduE::sfGFP* cloned into pBAD/*Myc*-His at NcoI and HindIII sites; Ap^R	This study
pBAD-*VE*	*pduV::mCherry-pduE::sfGFP* cloned into pBAD/*Myc*-His at NcoI and HindIII sites; Ap^R	This study
pBAD-*pduCDE-sfGFP*	*pduCDE::sfGFP* cloned into pBAD/*Myc*-His at NcoI and HindIII sites; Ap^R	This study
pBAD-*pduGH-sfGFP*	*pduGH::sfGFP* cloned into pBAD/*Myc*-His at NcoI and HindIII sites; Ap^R	This study
pBAD-*pduE-sfGFP*	*pduE::sfGFP* cloned into pBAD/*Myc*-His at NcoI and HindIII sites; Ap^R	This study
pBAD-*pduA-sfGFP*	*pduA::sfGFP* cloned into pBAD/*Myc*-His at NcoI and HindIII sites; Ap^R	This study
pXG10-SF	Plasmid served as the backbone for complementation experiments; pSC101* origin of replication; $P_{LtetO-1}$ promoter; Cm^R	[6]
pXG10-*pduB*	Plasmid for expression of PduB (M38A); Cm^R	This study
pXG10-*pduBB'*	Plasmid for expression of PduBB'; Cm^R	This study
pXG10-*pduM*	Plasmid for expression of PduM; Cm^R	This study
pXG10-*pduK*	Plasmid for expression of PduK; Cm^R	This study
pXG10-*pduJ*	Plasmid for expression of PduJ; Cm	This study

Supplementary Table 2. ssDNA Oligonucleotides used in this study.

Primers	Sequence (5'→3')
pduA_del_F	TCTTATAGTCCCAACTATCGGAACACTCCATGCGAGGTCTTTATGGTGTAGGCTGGAGCTGCTTC
pduA_del_R	GTTCCACCAGCTCATTGCTGCTCATTGGCTAATTCCCTTCGGTAACATATGAATATCCTCCTTAG
pduA_up	AAATATTGCACAAGCCAACTTATC
pduA_down	GGCCCAGGGTATCGCCAATG
pduA_sfGFP_F	CCTCACACCGATGTAGAAAAAATCTTACCGAAGGGAATTAGCCAAGGATCCGCTGGCTCCGCTGC
pduA_sfGFP_R	TCTGTTCCACCAGCTCATTGCTGCTCATTGGCTAATTCCCTTCGGGTGTAGGCTGGAGCTGCTTC
pduB1-37_del_F1	AGGGATAACAGGGTAATCTGAATTGCACAAGCCAACTTATCAATTTCGA
pduB1-37_del_R1	CCGTCTCTCGTATAGGTTGTCAGCTCATTGGCTAATTCCC
pduB1-37_del_F2	GGGAATTAGCCAATGAGCTGACAACCTATACGAGAGACGG
pduB1-37_del_R2	CCTGCAGGTCGACTCTAGAGGATCACTTCAGCGCGGTATCGGCC
pduB_up	TACACGTCATCCCACGCCCT
pduB_down	GTATCGCGCGGCAGCTCAAT
pduB M38A-F1	AGGGATAACAGGGTAATCTGAATTCTGATGCTCAACAGCAAGTC
pduB M38A-R1	AAACTGCAGCTTTTTTCTGCAGCAGCCGTCTCTCGTATAG
pduB M38A-F2	CTATACGAGAGACGGCTGCTGCAGAAAAAAGCTGCAGTTT
pduB M38A-R2	CCTGCAGGTCGACTCTAGAGGATCACTTCAGCGCGGTATCGGCC
pduJ_del_F	CCCTTTCGGGATCTCCATGCTTAATCACAGGAGAACGGCAGTATGGTGTAGGCTGGAGCTGCTTC
pduJ_del_R	GCGGTGCTCCTTATTCGCCATCGATTAGGCTGATTTCGGCATATGAATATCCTCCTTAG
pduJ_up	GATCGACACTCGCTGGTCGT
pduJ_down	CCTGAACGGAGGCCACATCA
pduK_del_F	GATGTTGAGGCCATTTTACCGAAATCAGCCTAATCGATGGTGTAGGCTGGAGCTGCTTC
pduK_del_R	GCAGAAGCTCTTTATCCATTACGCTTCACCTCGCTTGCCCATATGAATATCCTCCTTAG
pduK_up	TCATGGTTCGCGGCGATGTC
pduK_down	GATGGGATGGCCGGGAAACA
pduM_del_F	CCCGCATGCCTTTGCCCGGCTGGTAGGCCCGCGATGAACGTGTAGGCTGGAGCTGCTTC
pduM_del_R	CGTGACTCGTGCCAGATGCATGATTTACTCCTGCTTAATCATATGAATATCCTCCTTAG
pduM_up	GCGGGCTGATTTTCAACAAC
pduM_down	GCCGCTGAGCAAAACCAGTT
pduN_del_F	GCCAATGCGCGGAATATTCAATTAATTAAGCAGGAGTAAATCATGGTGTAGGCTGGAGCTGCTTC
pduN_del_R	GCCAGCGTCACCTGTTCGGGTATAAATCGCCATAACCGCCCCTTACATATGAATATCCTCCTTAG
pduN_up	TGCCGCTGGTATTCACCGAT
pduN_down	CTGCTGGATGGCCTCGAGTA
pduCDE_del_F	CGTCCGTCCTACATCTGATACCCACGAGGCTGATTCATGGTGTAGGCTGGAGCTGCTTC
pduCDE_del_R	GCCAGCTATATATCGCATACGAAATCCTTAATCGTCGCCCATATGAATATCCTCCTTAG
pduCDE_up	CGGCACCAGCTTTAGTAACG
pduCDE_down	TCCTGAATGCCGAACACGTT
pduCDEGH_del_R	TCCCAGTGCGTTATTCATACTGCCGTTCTCCTGTGATTACATATGAATATCCTCCTTAG
pduL_del_F	GCTTTGCATTCATTCCGGCAAGCGAGGTGAAGCGTAATGCATATGAATATCCTCCTTAG
pduL_del_R	GCAGGGTTTCGCCGTTCATCGCGGGCCTACCAGCCGGGCGTGTAGGCTGGAGCTGCTTC
pduL_up	CCTGAGCCTGAAGCGTCAG
pduL_down	GCAGGTCGATGAGCAGGAT

pduO_del_F	GGCATTGTAGATACGCTTTCGTGTTAAGGGGCGGTTATGGTGTAGGCTGGAGCTGCTTC
pduO_del_R	CGAGTTCAGAAGTATTCATTGATGAGTTCCCACGTTAATCATATGAATATCCTCCTTAG
pduO_up	ATGAAGTGGCCGTGGACT
pduO_down	AGCGGGCACTGCTGATAAC
pduP_del_F	CGCCATCGCGGCTATTAACGTGGGAACTCATCAATGAATGTGTAGGCTGGAGCTGCTTC
pduP_del_R	GTAGTGAGAAGGTATTCATCGCGACCTCAGTTAGCGAATCATATGAATATCCTCCTTAG
pduP_up	GCTGAGCGATGTCGTTCA
pduP_down	GACGCTGATGCGGTTATCTG
pduOPQS_del_R	TTCCTATAGCCTGAGACATGGTTAACCTCTTACAACAGTCATATGAATATCCTCCTTAG
pduOPQS_down	TGCTTCGCTCGGCGTGATGG
pXG10-F	TCTAGAGGCATCAAATAAAACGAAAG
pXG10-R	ATGCATGTGCTCAGTATCTCTATCAC
pXG10-pduB-F	GTGATAGAGATACTGAGCACATGCATTGTAGAAAAAATCTTACCGAAGGGAATTAGCC
pXG10-pduB-R	CTTTCGTTTTATTTGATGCCTCTAGATCAGATGTAGGACGGACGATCG
pXG10-pduM-F	GTGATAGAGATACTGAGCACATGCATCCCGCATGCCTTTGCCCG
pXG10-pduM-R	CTTTCGTTTTATTTGATGCCTCTAGATTACTCCTGCTTAATTAATTGAATATTCCG
pXG10-pduK-F	GTGATAGAGATACTGAGCACATGCATGTTGAGGCCATTTTACCGAAAT
pXG10-pduK-R	CTTTCGTTTTATTTGATGCCTCTAGATTACGCTTCACCTCGCTTGC
pXG10-pduJ-F	GTGATAGAGATACTGAGCACATGCATGATCTCCATGCTTAATCACAGGAG
pXG10-pduJ-R	CTTTCGTTTTATTTGATGCCTCTAGATTAGGCTGATTTCGGTAAAATGGC
pBAD-CDE-sfGFP-F	GGGCTAACAGGAGGAATTAACCATGAGATCGAAAAGATTTGAAGCAC
pBAD-CDE-sfGFP-R	GAGATGAGTTTTTGTTCTACGTATTATTTGTAGAGCTCATCCATGCC
pBAD-GH-sfGFP-F1	GGGCTAACAGGAGGAATTAACCATGCGATATATAGCTGGCATTGACA
pBAD-GH-sfGFP-R1	GGAGCCAGCGGATCCAGCATGGAGATCCCGA
pBAD-GH-sfGFP-F2	ATGCTGGATCCGCTGGCTCCG
pBAD-GH-sfGFP-R2	GAGATGAGTTTTTGTTCTACGTAAGCTT
pBAD-EA-F1	GGGCTAACAGGAGGAATTAACCATGAATACCGACGCAATTGAATCG
pBAD-EA-R1	AGAACCAGCAGCGGAGCCAGCGGATCCATCGTCGCCTTTGAGTTTTTTA
pBAD-EA-F2	CTGGCTCCGCTGCTGGTTCTGGCGAATTCGTGAGCAAGGGCGAGGAG
pBAD-EA-R2	CTCCTGTTAGCCCCTACTTGTACAGCTCGTCCATGCCGCC
pBAD-EA-F3	CAAGTAGGGGCTAACAGGAGGAATTAACCATGCAACAAGAAGCACTAGG
pBAD-EA-R3	GAGATGAGTTTTTGTTCTACGTATTATTTGTAGAGCTCATCCATGCC
pBAD-PA-F1	GGGCTAACAGGAGGAATTAACCATGAATACTTCTGAACTCGAAACCCTGATTCGCA
pBAD-PA-R1	CTTGCTCACGAATTCGCCAGAACCAGCAGCGGAGCCAGCGGATCCGCGAATAGAAAAGCC
pBAD-PA-F2	CTGGCGAATTCGTGAGCAAG
pBAD-PA-R2	CTGAGATGAGTTTTTGTTCTACGTA
pBAD-PE-F1	GGGCTAACAGGAGGAATTAACCATGAATACTTCTGAACTCGAAACCCTGATTCGCA
pBAD-PE-R1	CTTGCTCACGAATTCGCCAGAACCAGCAGCGGAGCCAGCGGATCCGCGAATAGAAAAGCC
pBAD-PE-F2	CTGGCGAATTCGTGAGCAAGGGCGA
pBAD-PE-R2	TTGCGTCGGTATTCATGGTTAATTCCTCCTGTTAGCCCC
pBAD-PE-F3	AACCATGAATACCGACGCAATTGAATCGATGGTCC
pBAD-PE-R3	GAGATGAGTTTTTGTTCTACGTATTATTTGTAGAGCTCATCCA
pBAD-J-F1	GGGCTAACAGGAGGAATTAACCATGAATAACGCACTGGGACTGGTTG
pBAD-J-R1	CTTGCTCACGAATTCGCCAGAACCAGCAGCGGAGCCAGCGGATCCGGCTGATTTCGGTAA
pBAD-GE-F1	GGGCTAACAGGAGGAATTAACCATGCGATATATAGCTGGCATTGACATCGGTAAC

pBAD-GE-R1	CTTGCTCACGAATTCGCCAGAACCAGCAGCGGAGCCAGCGGATCCCTGTCCATGCGCAAA
pBAD-LE-F1	GGGCTAACAGGAGGAATTAACCATGGATAAAGAGCTTCTGCAATCAACGGTC
pBAD-LE-R1	CTTGCTCACGAATTCGCCAGAACCAGCAGCGGAGCCAGCGGATCCTCGCGGGCCTACCAG
pBAD-OE-F1	GGGCTAACAGGAGGAATTAACCATGGCGATTTATACCCGAACAGGTGACG
pBAD-OE-R1	CTTGCTCACGAATTCGCCAGAACCAGCAGCGGAGCCAGCGGATCCTTGATGAGTTCCCAC
pBAD-QE-F1	GGGCTAACAGGAGGAATTAACCATGAATACCTTCTCACTACAAACGCGGTTGTA
pBAD-QE-R1	CTTGCTCACGAATTCGCCAGAACCAGCAGCGGAGCCAGCGGATCCTAGCAGTTCCTCCAG
pBAD-SE-F1	GGGCTAACAGGAGGAATTAACCATGAGCACCGCCATCAACAGCGTT
pBAD-SE-R1	CTTGCTCACGAATTCGCCAGAACCAGCAGCGGAGCCAGCGGATCCACCTCTTACAACAGT
pBAD-VE-F1	GGGCTAACAGGAGGAATTAACCATGAAGCGTTTGATGTTTATCGGCCCCA
pBAD-VE-R1	CTTGCTCACGAATTCGCCAGAACCAGCAGCGGAGCCAGCGGATCCTTTTGTAAGACATAA
pBAD-K-F1	GGGCTAACAGGAGGAATTAACCATGGCGAATAAGGAGCACCGCGT
pBAD-K-R1	CTTGCTCACGAATTCGCCAGAACCAGCAGCGGAGCCAGCGGATCCCGCTTCACCTCGCTT
pBAD-M-F1	GGGCTAACAGGAGGAATTAACCATGAACGGCGAAACCCTGCAGCGCA
pBAD-M-R1	CTTGCTCACGAATTCGCCAGAACCAGCAGCGGAGCCAGCGGATCCCTCCTGCTTAATTAA
pBAD-N-F1	GGGCTAACAGGAGGAATTAACCATGCATCTGGCACGAGTCACGGG
pBAD-N-R1	CTTGCTCACGAATTCGCCAGAACCAGCAGCGGAGCCAGCGGATCCACACGAAAGCGTATC
pBAD-T-F1	GGGCTAACAGGAGGAATTAACCATGTCTCAGGCTATAGGAATTTTAGAACTCACCA
pBAD-T-R1	CTTGCTCACGAATTCGCCAGAACCAGCAGCGGAGCCAGCGGATCCCCCCTCCACCATCTG
pBAD-U-F1	GGGCTAACAGGAGGAATTAACCATGGAAAGACAACCGACAACGGATCGC
pBAD-U-R1	CTTGCTCACGAATTCGCCAGAACCAGCAGCGGAGCCAGCGGATCCCGTCCGGGTGATCGA
pBAD-B-F1	GGGCTAACAGGAGGAATTAACCATGAGCAGCAATGAGCTGGTGGAAC
pBAD-B-R1	CTTGCTCACGAATTCGCCAGAACCAGCAGCGGAGCCAGCGGATCCTCAGATGTAGGACGG
pBAD-B’-F1	GGGCTAACAGGAGGAATTAACCATGGCAGAAAAAAGCTGCAGTTTAACGG

Supplementary Table 3. The distribution of the number of "Shell first", "Cargo first", and "Concomitant" events in PduE-mCherry/ PduA-sfGFP and PduE-mCherry/ PduA-sfGFP assembly. Note: The "Concomitant" event reported here either represents concomitant assembly, or a fusion following a shell/cargo independent assembly event.

	**Shell first**	**Cargo first**	**Concomitant**
PduE-mCherry/PduA-sfGFP	71	72	175
PduJ-mCherry/PduE-sfGFP	75	74	178

Supplementary Table 4. Diffusion coefficient and mobile fractions of Pdu MCP per cell measured using FRAP. *n* represents the number of cells.

	**Mobile fraction (%)**	**Diffusion coefficient (D, × 10^{-4} $\mu m^2 \cdot s^{-1}$)**	**Half Life ($\tau_{1/2}$, s)**
PduE (cargo)	83 ± 6 (n = 20)	4.02 ± 1.89 (n = 20)	323 ± 171 (n = 20)
PduA (shell)	6 ± 3 (n = 19)	0.28 ± 0.15 (n = 19)	485 ± 407 (n = 19)
PduGH (free)	96 ± 4 (n = 15)	N/A	1.2 ± 0.4 (n = 15)

Supplementary References

1. Zinder ND, Lederberg J. Genetic exchange in *Salmonella*. *Journal of Bacteriology* **64**, 679 (1952).
2. Simon R, Priefer U, Pühler A. A broad host range mobilization system for in vivo genetic engineering: transposon mutagenesis in gram negative bacteria. *Biotechnology* **1**, 784-791 (1983).
3. Datsenko KA, Wanner BL. One-step inactivation of chromosomal genes in Escherichia coli K-12 using PCR products. *Proc. Natl. Acad. Sci. U.S.A* **97**, 6640 (2000).
4. Koskiniemi S, Pränting M, Gullberg E, Näsvall J, Andersson DI. Activation of cryptic aminoglycoside resistance in *Salmonella enterica*. *Molecular Microbiology* **80**, 1464-1478 (2011).
5. Martínez‐García E, de Lorenzo V. Engineering multiple genomic deletions in Gram‐negative bacteria: analysis of the multi‐resistant antibiotic profile of *Pseudomonas putida* KT2440. *Environmental Microbiology* **13**, 2702-2716 (2011).
6. Corcoran CP, Podkaminski D, Papenfort K, Urban JH, Hinton JC, Vogel J. Superfolder GFP reporters validate diverse new mRNA targets of the classic porin regulator, MicF RNA. *Molecular Microbiology* **84**, 428-445 (2012).
