# Supplementary material for: Biogenesis of a bacterial metabolosome for propanediol utilization
Source: Nat Commun. 2022 May 25;13:2920. doi: 10.1038/s41467-022-30608-w (PMC9132943; doi:10.1038/s41467-022-30608-w)
Supplement: Supplementary file 3 — Reporting Summary [file 41467_2022_30608_MOESM3_ESM.pdf]

nature research

Corresponding author(s): Professor Luning Liu

Last updated by author(s): Apr 14, 2022

Reporting Summary

Nature Research wishes to improve the reproducibility of the work that we publish. This form provides structure for consistency and transparency in reporting. For further information on Nature Research policies, see our Editorial Policies and the Editorial Policy Checklist.

Statistics

For all statistical analyses, confirm that the following items are present in the figure legend, table legend, main text, or Methods section.

n/a	Confirmed	
☐	☒	The exact sample size (*n*) for each experimental group/condition, given as a discrete number and unit of measurement
☐	☒	A statement on whether measurements were taken from distinct samples or whether the same sample was measured repeatedly
☒	☐	The statistical test(s) used AND whether they are one- or two-sided<br>*Only common tests should be described solely by name; describe more complex techniques in the Methods section.*
☒	☐	A description of all covariates tested
☐	☒	A description of any assumptions or corrections, such as tests of normality and adjustment for multiple comparisons
☐	☒	A full description of the statistical parameters including central tendency (e.g. means) or other basic estimates (e.g. regression coefficient) AND variation (e.g. standard deviation) or associated estimates of uncertainty (e.g. confidence intervals)
☒	☐	For null hypothesis testing, the test statistic (e.g. *F*, *t*, *r*) with confidence intervals, effect sizes, degrees of freedom and *P* value noted<br>*Give P values as exact values whenever suitable.*
☒	☐	For Bayesian analysis, information on the choice of priors and Markov chain Monte Carlo settings
☒	☐	For hierarchical and complex designs, identification of the appropriate level for tests and full reporting of outcomes
☐	☒	Estimates of effect sizes (e.g. Cohen's *d*, Pearson's *r*), indicating how they were calculated

Our web collection on statistics for biologists contains articles on many of the points above.

Software and code

Policy information about availability of computer code

Data collection	Transmission electron microscopy (TEM) data collected by a Gatan Rio 16 camera and DigitalMicrograph software. Confocal microscopy used ZEISS ZEN microscope software to run the confocal and collect images.
Data analysis	Confocal microscopy data was analyzed with ImageJ-win64. Colocalisation analysis were performed by using the Coloc2 plugin in ImageJ to generate Pearson's correlation coefficient R and scatterplots. Simulated fluoresce curves of the FRAP experiment were generated on SigmaPlot14 software to estimate the diffusion coefficient. The protein sequences of the genomes were produced using Prokka v1.14.6. The Pdu protein sequences were queried against the proteomes using BLASTp v2.5.0+ with a threshold of 90% amino acid identity. To build the phylogenetic tree of the 61 bacterial genomes, the universal single-copy orthologs of Gammaproteobacteria were obtained from each genome using BUSCO v5.2.2. From the BUSCO result, 327 genes were found to be present in all the genomes. Each gene set was aligned with Mafft v7.475. The alignments were concatenated with SeqKit v0.15.0, then trimmed with Trimal v1.4 with the automatic method. A phylogenetic tree was constructed from the alignment with Fasttree v2.1.10 using the JTT+Gamma model. The structure of PduB1-37, PduM, and PduK proteins was predicted by AlphaFold2, accessed via ColabFold. The structures were visualized in ChimeraX1.3. The conservation values were automatically calculated by ChimeraX1.3 used the entropy-based measure from AL2CO.

For manuscripts utilizing custom algorithms or software that are central to the research but not yet described in published literature, software must be made available to editors and reviewers. We strongly encourage code deposition in a community repository (e.g. GitHub). See the Nature Research guidelines for submitting code & software for further information.

Data

Policy information about availability of data

All manuscripts must include a data availability statement. This statement should provide the following information, where applicable:

- Accession codes, unique identifiers, or web links for publicly available datasets
- A list of figures that have associated raw data
- A description of any restrictions on data availability

All data needed to evaluate the conclusions in the paper are present in the main text or the supplementary materials. The source data underlying Figs. 2c, 2d, 3c, 3g, 4c, 4g, 5a, 5b, 5e, 6g, Supplementary Figs. 6a, 7, 10c, 11, and 15 are provided as a Source Data file. The complete bacterial genomes were downloaded from the Refseq bacterial database according to https://www.ncbi.nlm.nih.gov/genome/doc/ftpfaq/.

Field-specific reporting

Please select the one below that is the best fit for your research. If you are not sure, read the appropriate sections before making your selection.

☒ Life sciences ☐ Behavioural & social sciences ☐ Ecological, evolutionary & environmental sciences

For a reference copy of the document with all sections, see nature.com/documents/nr-reporting-summary-flat.pdf

Life sciences study design

All studies must disclose on these points even when the disclosure is negative.

Sample size	Sample sizes were not predetermined based on statistical methods, but were chosen according to the standards of the field (at least three independent biological replicates for each condition), which generated sufficient statistics for analysis. Twenty bacterial cells dual-labelled with fluorescence proteins were selected for colocalisation analysis, which is sufficient to calculate the Pearson's correlation coefficient R.
Data exclusions	Representative high quality confocal and TEM images were shown in the paper. Low quality and low resolution images were excluded, which is standard practice for TEM and confocal imaging and can be considered as pre-established criteria.
Replication	All of the experiments were repeated more than three times, and were reproduced successfully.
Randomization	Bacterial colonies were selected randomly for cell culture, growth assays, and confocal/TEM imaging. Fluorescently tagged bacterial cells were selected randomly for colocalisation analysis. All samples/organisms were allocated into experimental groups randomly.
Blinding	Investigators were not blinded. Blinding was not possible because sample preparation and data collection were conducted by the same investigators. Blinding during collection was not needed because conditions were well controlled. Blinding is also not necessary because the results are quantitative and did not require subjective judgment or interpretation.

Reporting for specific materials, systems and methods

We require information from authors about some types of materials, experimental systems and methods used in many studies. Here, indicate whether each material, system or method listed is relevant to your study. If you are not sure if a list item applies to your research, read the appropriate section before selecting a response.

Materials & experimental systems

n/a	Involved in the study	
☒	☐	Antibodies
☒	☐	Eukaryotic cell lines
☒	☐	Palaeontology and archaeology
☒	☐	Animals and other organisms
☒	☐	Human research participants
☒	☐	Clinical data
☒	☐	Dual use research of concern

Methods

n/a	Involved in the study	
☒	☐	ChIP-seq
☒	☐	Flow cytometry
☒	☐	MRI-based neuroimaging
